# A developmental pathway toward leadership for educational change: the Educators’ experiences of the educational scholar program

**DOI:** 10.1186/s12909-023-04015-8

**Published:** 2023-01-16

**Authors:** Fatemeh Keshmiri

**Affiliations:** 1 Medical Education Department, Education Development Center, Shahid Sadoughi University of Medical Sciences, Yazd, Iran; 2 School of Public Health, Shahid Sadoughi University of Medical Sciences, Yazd, Iran

**Keywords:** Leadership, Scholarship, Change leadership, Scholarship of teaching and learning, Faculty development, Educational change, Educational scholar program, Teaching scholar program

## Abstract

**Introduction:**

The Educational Scholar Program (ESP) is a creative method to focus on the quality of education and the scholarship of education. This paper critically investigated how educational educators perceived the Educational Scholar Program.

**Method:**

The ESP was developed according to the project-based learning method. An interdisciplinary strategy was used by participating educators from different schools, including nursing, medicine, public health, dentistry, and pharmacy. (*n* = 27). Semi-structured interviews explored the experiences of the participants in the ESP. A conventional content analysis approach introduced by Graneheim and Lundman was used for data analysis.

**Results:**

A theme of “a developmental pathway toward leadership for educational change” was explored into four categories: “Motivated for educational change,” “collaborative learning through the application of knowledge,” “tensions of change agent,” and “a perceived responsibility of a change agent.”

**Conclusion:**

The participants perceived the ESP as a platform for improving leadership capabilities for educational change through the Scholarship of Teaching and Learning (SoTL). Using the ESP as a faculty development program to train educational leadership for change is suggested.

**Supplementary Information:**

The online version contains supplementary material available at 10.1186/s12909-023-04015-8.

## Background

Educators and academics were recognized as crucial factors in driving educational change and improving university quality [1, 2]. The educators were encouraged to involve in activities of scholarship of teaching and learning (SoTL) to respond to the growing change in universities [3]. The SoTL activities were introduced as a new means to empower educators and a strategy for institutional improvement in teaching and learning [4].

The SoTL defines research/scholarship as a rigorous examination of teaching and learning by educators who actively participated in the educational process [5]. Educators in the SoTL activities could improve the quality of education by resolving educational problems or applying an innovative method/approach in the educational community [6, 7]. The main elements of SoTL included literature-informed, theoretically grounded, and methodologically rigorous research in education contexts [8]. Beckman and colleagues introduced a three-step scholarly approach that included the refinement of the study question and objectives by studying a conceptual framework, reviewing the literature and finding existing gaps in the community (step 1), developing appropriate study designs and methods (step 2), and select outcomes (distinguishing from assessment methods and instruments, and levels of Kirkpatrick's hierarchy of reaction, learning, behavior, and results) (Step 3) [9].

Glassick described six criteria of SoTL, including clear goals, preparation, appropriate methodology, significant results, effective presentation, and reflective critique [9]. The SoTL areas consist of teaching, educational planning, counseling, learner evaluation, management, and educational leadership [10, 11].

The educators needed to acquire the competencies of educational scholarship as a critical competency for responding to growing changes, adapting to innovations in technology and approaches, achieving excellence in education, and directing change in the institute [12–15].

An educational scholar program (ESP) as a faculty development program has been introduced that focuses on improving educators' capabilities in the SoTL activities [16]. The main goal of ESP was to the professional development of educators by enhancing their experience in designing, implementing, and leading a SoTL as an educational change project [6, 7, 12, 16–19]. Literature in the medical education field contains reports on ESPs at several universities: Harvard, USCF, McGill, UCLA, University of Michigan, University of Washington, and Johns Hopkins [16]. Regarding expanding ESP as a faculty development program, further studies are required to explain the various dimensions of ESP in the educational community [20].

In the investigated context, the empowerment programs were mainly conducted through workshops focusing on improving knowledge about teaching and learning methods [21]. The prototypical faculty development programs were short courses involving workshops on educational principles and teaching–learning techniques. The evaluation of the workshops was conducted by assessing participants’ satisfaction and self-efficacy [22]. In the present study, for the first time, the ESP as a faculty development program was conducted in the investigated context and was explored from the participants’ viewpoints.

This paper critically investigates how educators perceived the ESP as faculty development for educational change. The main research question was how the participants perceived the components of ESP (antecedents, process, and outcomes).

### The educational scholar program (ESP)

#### Development of ESP

The ESP was developed by assessing the need of stakeholders (educators, directors, students, and administrations), reviewing the institute’s mission and programs, and reviewing literature about ESP in different universities. The extracted results were discussed in the expert panel sessions where 12 experts in health profession education participated. The components of the ESP include the educational objectives, educational strategy (interdisciplinary strategy,) teaching–learning method (project-based learning), and assessment methods developed in the expert panel.

#### The objective of ESP

Improving the participants’ competencies in the scholarship of teaching and learning projects was defined as a primary objective of ESP. An educator in SoTL activity requires a systematic approach to solve a problem or improve a process in the educational context [23]. In this regard, the participants were asked to design, implement, and evaluate a SoTL project in their educational communities and disseminate their results.

#### The educational strategy of ESP

A interdisciplinary strategy was used; educators from different schools, including nursing, medicine, public health, dentistry, and pharmacy, participated.

#### Teaching and learning method of ESP

The program was designed according to the project-based learning method. The steps of project-based learning include 1) beginning inquiry (asking questions, formulating goals, planning procedures, and designing investigations), 2) directing inquiry (developing data collection, conducting data searches, and constructing methods), 3) analysis and critical reflection (analyzing data, drawing conclusions, collaborating on written work), and 4) dissemination knowledge and feedback-seeking (giving and seeking feedback) [24, 25]. Project base learning provides structured situations for practical learning. Participants need to learn different competencies in SoTL as a complex concept by active involvement and reflection in the structured steps.

#### Content of ESP

The common educational topics of ESPs included educational models and learning theories, educational research and scholarship, educational change leadership capabilities, teaching skills, educational strategy and teaching–learning methods, curriculum development and evaluation, and student assessment methods.

#### Structure of ESP

The ESP was designed as a long course in two phases, including training and an educational scholarship. We have described the ESP steps in the educational scholarship phase in Table 1.

- In the training phase, educators were helped to familiarize themselves with educational theories, models, and principles in the domains such as curriculum development, teaching–learning methods, evaluation, and assessment. In this phase, they experienced learning in small group settings, self-directed learning, and reflection assignments.
- In the educational scholarship phase, the participants experienced a project-based learning method in the interdisciplinary team [24, 25]. This method directs structured steps from the beginning of idea generation to dissemination. As well, the steps of project-based learning are well-matched with SoTL stages. The first step of the present program was compatible with the first step of Beckman's model including a conceptual framework, reviewing the literature, and exploring needs in the community [26]. The step of 'directing inquiry' is similar to the second and third steps of Beckman's Model including the development of appropriate study designs and methods and outcomes [26].
- A mentor who was an expert in health professions education directed all steps of the SoTL process from design to implementation and dissemination.

**Table 1:** The ESP steps in the educational scholarship phase

Beginning inquiry	*Idea and problem identification*
	- The participants were asked to find their problem in their context by conducting a need assessment plan - The problem was explained in consultation with peers and after reviewing the literature -The participants require creating a conceptual framework, reviewing the literature, and finding gaps in understanding -They refined their intent to solve a recognized problem or apply an innovative method in their educational community
Directing inquiry	*Study design*:
	- The participants identified appropriate study designs and methods for their intent - The participants were asked to formulate a SoTL proposal for their idea - The proposals were reviewed and finalized by a panel of experts. The participants applied for a grant from a university or national agency
	*Preparation and implementation*:
	- The participants formed a team of peers and collaborators to conduct the project and attain preparedness to conduct the SoTL - Support sources such as resources, educational materials, equipment, and organizational support were prepared - The implementation of the SoTL project was conducted according to the designed plan
Analysis and critical reflection	- The team members and their mentors participated actively in the critical reflective meeting during the SoTL phases - The results of the reflection and feedback meetings were used to remedy or improve the SoTL process and outcomes
Dissemination and feedback-seeking	- The results of the SoTL project were disseminated among colleagues for feedback-seeking and giving critique - The results of SoTL were published as educational scholarship articles in journals and congress to share the participants’ experiences of their SoTL

#### Assessment

The participants' performance from designing to disseminating the SoTL project was assessed. The evaluation of the SoTL projects was conducted according to the Glassick criteria, including clear goals, appropriate methods, adequate preparation, effective presentation, significant results, and reflective critique [9].

## Method

This study was designed from a paradigmatic interpretive perspective, which considered with social realities as constructed and valuing individual, subjective experience with [27]. In this perspective, the research emphasizes the experiences of people who contributed and experienced a situation/phenomenon and takes a qualitative approach to data collection [27]. The qualitative methods help explore the ESP's different aspects from the perspective of participants who attended the program.

The present study used qualitative content analysis. The content analysis approach is suitable when new areas are to be investigated in an exploratory manner or if it has been decided to explore a known area from a fresh perspective [28]. The experiences of participants related to ESP as a complex phenomenon that needs to be investigated in an exploratory manner. This approach allows new insights to emerge for explaining the ESP [29].

Three cohorts of the ESP were conducted at Shahid Sadoughi University of Medical Sciences from 2018 to 2020. Thirty-six educators participated in the cohorts of the ESP.

### Demographic information

Inclusion criteria were educators who completed the ESP course and conducted at least a SoTL project. The educators with different working experiences and academic ranks participated in the ESP. The participants in different groups of gender, age, work experience, and conduction SoTL project in different areas (e.g., teaching–learning, curriculum development, assessment, and evaluation) were contributed by a maximum variation sampling. Twenty-seven educators in different faculties of Shahid Sadoughi University of Medical Sciences participated in the study. Table 2 are showed the demographic characteristics of the educators.

**Table 2:** The demographic characteristic of participants

**Gender n(%)**	
Male	12 (44.44%)
Female	15 (55.55%)
**School of participants n(%)**	
Dentistry	8 (29.62)
Public Health	5 (18.51)
Medicine	5 (18.51)
Pharmacy	2 (7.40)
Nursing and Midwifery	7 (25.92)
**Academic degree n(%)**	
Preceptor	2 (7.40)
Assistant Professor	14 (51.85)
Associate Professor	7 (25.92)
Full Professor	4 (14.81)
**Working experience (mean ± SD)**	8 ± 4

### Data collection

Data were collected using individual and semi-structured interviews. The interview guide was developed and considered the main components of ESP, such as antecedent, process, and outcomes. A pilot interview was conducted to test the interview guide. Interviews started with open-ended questions and continued with probing questions. (Appendix 1). A trained interviewer performed the interviews. Each interview lasted between 60–90 min. All interviews were recorded during the data collection. The data collection process continued until a rich interpretation was obtained, and no new code emerged during the interviews (saturation of results). The data were collected and analyzed in Persian and then translated into English for this paper. The results were translated and back-translated to English to ensure accuracy.

### Data analysis

Data were analyzed by the conventional content analysis approach introduced by Graneheim and Lundman [30]. All recorded interviews were transcribed verbatim immediately after the interview, and the transcripts were reviewed several times. The meaning units were extracted from the participants’ words and expressions, reflecting their experiences. After that, open coding was generated by taking notes in the margins of the text. At this stage, we transferred the codes to coding sheets. These codes were then grouped into categories. Finally, the theme emerged by comparing and contrasting the categories.

In this study, two qualitative researchers performed the data coding in a qualitative study. They were MS.c and Ph.D. in health professions education and the mean years of working experience in the field of qualitative research were 5 ± 1. The process was supervised by an expert who had expertise in qualitative research. In cases of disagreement over the coding, discussions about the codes were continued until a consensus was achieved.

### Rigor

In this study, the criteria described by Schwandt [31] were used to ensure trustworthiness. The criteria include credibility, transferability, dependability, and confirmatory. The main techniques of credibility were achieved by prolonged engagement, peer debriefing, and member checks [31]. In the current study, semi-structured interviews, field notes, and lengthy engagement with the research topic were used to achieve the credibility of the data that was applied in the data collection, analysis, and interpretation steps. In addition, the extracted codes and categories were reviewed by the participants (member check), and the research team (peer check) was conducted to ensure the validation of the findings. Texts and related analyses were returned to the participants for member checking to ensure that codes and categories were consistent with what they had experienced. Peer checks and member checks were used in the steps of data collection, analysis, and interpretation. For dependability and confirmability, an external audit required in examining the process results in a dependability judgment, and the product (data and reconstructions) results in a confirmability judgment [31]. To do this, two experts in qualitative research checked the encoding process and forming of the categories (external audit). The external audit was conducted in the steps of data analysis and interpretation and reporting. Thick descriptive data is required for achieving the criteria of transferability [31]. In this study, the steps of research, especially the data analysis, have been thoroughly documented. The present study provides a clear description of the context, and participant characteristics, that are considered in the steps of data collection, data analysis, and reporting process to facilitate the transferability of the findings.

### Ethical consideration

The study was approved by the Ethics Committee at Shahid Sadoughi University of Medical Sciences, Yazd, Iran. (ID: IR.SSU.REC.1398.179). At the beginning of the interviews, the purpose of the research, the interview method, and the individuals’ right to participate or refuse to it in the study were explained. The participants were assured confidentiality of the recorded interviews and the collected information. Informed consent was obtained from them.

## Results

A theme was explored as “a developmental pathway toward leadership for educational change” into four categories: “motivated for educational change,” “collaborative learning through the application of knowledge, “tensions of change agent,” and “a perceived responsibility of a change agent.” (Table 3).

**Table 3:** The experiences of participants of the education scholar program

Sub-Category	Category	Theme
Understanding the need for educational change	Motivated for educational change	**Developmental pathway toward leadership for educational change**
Recognizing personal limitations		
Involvement in reflective and collaborative learning	Collaborative learning through the application of knowledge	
Knowledge transformation		
Resistance to change	Tensions of change agent	
Resource limitation		
Enhancing self-efficacy belief as a change agent	A Perceived responsibility as a change agent	
Promoters of accountability in educational change		

### A developmental pathway toward leadership for educational change

The participants believed the design and implementation of the SoTL project in the ESP had prepared them to learn about educational leadership capabilities. The category of with motivated for educational change with was explored as an antecedent of ESP. The perceived process of ESP is explored in the categories; with collaborative learning through the application of knowledge”, and with tensions of change agent.” “A perceived responsibility of a change agent” as a perceived outcome was explored.

#### A- Motivated for educational change

The category explored two antecedent factors for pursuing the participants to contribute to the ESP. The participants stated that ESP improved their perception of the necessity of change in the educational process. Moreover, they believed personal limitations in solving their perceived needs motivated them to contribute to the ESP. The educational change meant the development of education in the educational community or the design and implementation of an effective method and strategy for solving an educational problem.

#### A.1- Understanding the need for educational change

The category addressed the factors encouraging participants to contribute to ESP. The perceived need to change in the educational process was explored as a motivating factor. A participant stated:

“I was not satisfied with the level of student's abilities at the end of the rotation. I want students to become more robust in terms of knowledge and practice. I realized there was a need to change my training, but I did not know how I could do it‘’ (Participant No. 3).

#### A.2- Recognizing personal limitations

The perception of personal and professional limitations, such as unfamiliarity with the practical education principles and theories and lack of capability to apply the educational strategies and methods explained in this category. A participant stated:

“Just because I had a board-certified, I became an educator. I did not know anything about the education process. I understand my defects and have a plan for self-development by the ESP.” (Participant No. 23).

#### B- Collaborative Learning through the application of knowledge

The participants’ experiences about their learning process as a scholar and leaders were explained in this category.

#### B.1- Involvement in reflective and collaborative learning

The involvement in collaborative learning to solve a problem was explored as the main element of the ESP process. A participant acknowledged:

“I had taken a series of notes in previous empowerment workshops and have a folder of these papers that I had taken and forgotten. This program was the first time I would discuss the experienced problems with our colleagues and pursue to resolve them.” (Participant No. 16).

Creating an opportunity for reflection on participants’ experiences and duties during formal or informal settings was explored in the category. The participants experienced concepts of reflection-in-action and on-action in the ESP. A participant stated:

“Once, a participant presented the challenges of her lesson in basic sciences and suggested a solution for it. I had not thought about the challenges before that. I thought about my class after the interdisciplinary discussion. I was motivated to change the teaching method in my class. I could find solutions for improving my teaching that had not already been thought about that”. (Participant No. 7).

The participants’ experiences related to interdisciplinary collaboration in conducting a scholarly project were explored. A participant stated:

“In the interdisciplinary group, the participants in different disciplines discussed their educational problems and exchanged their experiences. We extracted a list of educational problems and their solutions. We designed SoTL projects to solve them.” (Participant No. 16).

#### B.2- Knowledge transformation

The participants believed an opportunity to apply their knowledge in the SoTL project helped them to experience a deep learning process. A participant stated:

“I could understand better the components of education when I apply my knowledge in the SoTL.” (Participant No. 27).

“I found the ESP as a job market in my specialty field. Implementing my SoTL project helped me learn to lead an educational change in my class. Likewise, when I entered a hospital, I found the reality of nursing practice.” (Participant No. 14).

#### C- Tensions of change agent

The experienced tensions as a leader were categorized in the category. The resistance of colleagues and managers, the limitation of qualified human resources and equipment to implement SoTL projects, and the administrative challenges were explored as tensions in directing educational change projects.

#### C.1—Resistance to change

The participants were primarily faced with resistance from collaborators and administrators when presenting their SoTL idea for changing an education process, such as using interactive methods. A participant stated:

“In my opinion, the biggest problem about conducting a SoTL project was individuals’ resistance that often distracts me from my goal.” (Participant No. 5).

#### C.2- Resource limitation

The participants believed that although they understood the need for educational change and were highly motivated to address it, barriers such as human resources and educational equipment prevented the effective implementation of a SoTL project. A participant stated:

“In my project about student assessment, the main problem was the restriction of equipment for the OSCE such as simulation equipment in my school.” (Participant No. 11).

“We did not have enough computers to run electronic reasoning tests. We wanted to improve the quality of our examination, but we did not have enough infrastructure and faced many challenges.” (Participant No. 1).

#### D- A perceived responsibility of a change agent

The participants believed the ESP led them to perceive their responsibility in the educational change. The participants believed they developed their leadership skills for change and self-efficacy as change agents. The participants were also motivated to play as promotors of educational change in their institute.

#### D.1- Enhancing self-efficacy belief as a change agent

The participants’ experiences regarding the acquired leadership competencies for educational change were explored in the category. They believed conducting a SoTL process from planning to evaluation improved their self-efficacy for directing a project of educational change. A participant stated:

“I already knew that my teaching method require change, but we did not know what the right thing and the principles were. Now, I achieved the assertion to change because I know the right way. Moreover, I can empower my peer and teammate.” (Participant No. 18).

The participants believed they could enhance their sensitivity to learning problems and their capability in critical appraisal and designing and implementing a proper method to solve them. A participant stated:

“I critically reviewed my educational process. I found that the teaching method needed to be revised. I revised and focused on the adjustment of the components and selected appropriate methods from my toolbox.” (Participant No. 24).

“I recognized the midwifery final exam needs to be changed and brought out in my department. Now, we have changed the final exam based on what was taught.” (Participant No. 17).

#### D.2- Promotors of accountability in educational change

This category explained the participants’ experience as a promoter of educational change. The factors of self-motivation, such as receiving positive feedback from stakeholders and understanding the ability to change, motivated the participants as change agents. A participant believed:

“The important thing for me was the positive feedback of my students. They said, “your new method encourages us to learn deeply.” The new method motivated my students. As well, they being a motivating force for me.” (Participant No. 11).

The participants believed a perceived responsibility was encouraging colleagues to participate in educational change projects as promotors of change. They could persuade their colleagues to improve their training through SoTL. A participant stated:

“I encouraged my coworkers to collaborate on the SoTL projects. I talked to my collages about DOPs (Direct Observation procedural skill tests). All of them asked to know more; it was exciting that they were simulated how to improve the student assessment. After a while, they would come and want to get involved in the SoTL.” (Participant No. 19).

## Discussion

This study used ESP as faculty development for educational change. The participants’ experiences with the ESP have been explored as “a developmental pathway toward leadership for educational change.” the category of “motivated for educational change” was explored as an antecedent factor for involvement in the ESP and the SoTL. Understanding the need to change and recognizing personal limitations were explored as antecedent factors for involving in ESP. The participants acknowledged they had understood the challenges of education but could not find a suitable solution. They believed that personal limitations for solving the perceived problems more than any other factors motivated them to contribute to the ESP. The participants’ perceived weaknesses in applying the new methods in education and unfamiliarity with the education principles motivated them to contribute to the ESP. Similarly, Elmberger et al. [32] recognized personal motivation as a central factor at the individual level for engaging clinical educators in educational development activities. In line with our results, Knight and colleagues [33] emphasized the importance of intrinsic factors and professional motives in educators’ educational development.

The “collaborative learning through the application of knowledge” was categorized as the participant’s experiences of the learning process in the ESP. The category highlighted three components of the ESP: reflection, collaborative learning, and knowledge transformation in the educational community. The ESP provided a practical situation for analyzing the educational challenges, selecting the high-priority problem, and designing the scholarly project to solve them. The participants were asked to find a problem in their educational community based on the needs assessment plan. Then, they planned for educational change in a reliable method by reviewing the literature and the opinion of colleagues. They conducted a SoTL project to solve a perceived problem or improve an education process in their context. Finally, the participants evaluated the outcomes of the educational change project and published their findings. The formation of interdisciplinary teams for leading the SoTL project and reflection in small groups were explored as a positive experience of the ESP process. The participants believed the ESP enhanced their self-efficacy for change as a leader by providing a situation for practical learning, interdisciplinary interactions, and supportive mentoring. Likewise, Fanghanel et al. recognized the SoTL as a reflective methodology for the professional development of educators [17]. The participants acknowledged that applying their knowledge during the design and implementation of the SoTL project resulted in learning the critical competencies as change agents. Steinert et al. [19] stated that the opportunity to apply knowledge among participants led to a better understanding of the educational community, and facilitated change in their profession. The transformation of knowledge in the learning environment and leadership development for change among educators was recognized as an advantage of ESP [6, 22, 34]. Fields et al. explored “mentoring and empowering” and “action orientation” as critical characteristics of the development of educational leadership competency [35]. Likewise, in our ESP, a mentor facilitated and supervised the participants in the learning pathway through applying knowledge.

“Tension of the change agent” was addressed as a negative experience as a leader. The resistance to change among administrators and colleagues was the main challenge in SoTL from participants' viewpoints. In line with our findings, McGrath and colleagues explained the complex nature of the change process from the perspective of the change agents. They explored the 'change as overcoming resistance’ category that addressed the resistance of managers and colleagues [36]. Moreover, as change agents, the participants experienced systemic challenges, a lack of support from educational administrators and various stakeholders, and inadequate equipment and resources. The teacher-centered and discipline-based approaches in the investigated context may affect resource shortages such as facilities and qualified personnel. In line with our results, limitations of educational infrastructure, and available resources, were identified as barriers to conducting the SoTL project in different studies [1, 37–39]. Furthermore, the constraints of qualified colleagues, uncooperative administration, the limitation of peer support, and lack of motivation have been defined as the challenges of implementing SoTL projects [37, 40, 41] that were similar to the present findings.

The category of “a perceived responsibility of a change agent” was explored as the perceived outcome of the ESP. The participants believed that the ESP helped them become familiar with educational concepts and achieve sub-competencies of leadership and scholarship. They acknowledged their recognition of the responsibility of the leadership of an educational change improved. They achieved a new perspective on the educational process and enhanced their motivation for involving in the educational change process. Furthermore, they believed their reflective ability, critical appraisal, and self-efficacy belief as change agents improved. Likewise, Steinert and colleagues showed that the ESP facilitated enhancing a sense of belonging to the educational community and forming a sense of confidence in implementing change projects among the participants. Their results indicated that more than half of the participants in ESP played a leadership role in educational change in their department, which was called the unpredictable outcome of the ESP [19]. Macario and colleagues revealed that ESP consists of training sessions, and project implementation in the educational community improved the participants’ competency for conducting innovative teaching–learning processes in the investigated schools [42].

“Promotors of accountability in educational change” was explored as the experienced outcomes. The categories addressed the factors of the creation of motivation to contribute to a change project in the educational community. The participants, as change leaders, could persuade their colleagues to participate in SoTL. The steps of team formation, the implementation of SoTL, and presenting findings provided proper situations for persuading others to be involved in the change project. In addition, the participants stated positive outcomes and feedback from their learners as key motivating factors that kept them going through the SoTL as an educational change project. The feeling of self-efficacy in teaching and satisfaction were pleasure elements that some participants described as motivational factors. Similarly, Fields et al. indicated that change agents' attribute was others’ motivation to participate in and facilitate a change project in the teaching and learning process [35].

These results showed that creating opportunities according to the project-based learning in the empowerment program played an important role in developing the change leadership competencies of the educators. The ESP was perceived as a developmental pathway to develop change leadership skills. The participants learned the micro-skills of leadership in the design, implementation, evaluation, and dissemination of a SoTL as a change project. It is suggested that the impact of ESP in the educational community will examine by a longitudinal study in future studies. Also, the program evaluation based on Kirkpatrick's levels will be done using quantitative and qualitative methods in the next studies.

### Limitation

The study was conducted in one university. The results may explore the aspects of ESP from the participants’ viewpoints. The qualitative method limited the generalizability of the present results.

## Conclusion

The results showed that an antecedent factor of ESP was explained in the “Motivated for educational change” category, which addressed the role of motivation for change among participants. The category of “collaborative learning through the application of knowledge” as the learning cycle of the ESP focused on reflection, collaborative learning, and knowledge transformation in the educational community. The results also showed that although individual and motivational factors were influential in developing leadership for change, the participants faced tensions such as resistance to change of colleagues for conducting the educational change projects and constraint of resources. The experienced outcomes of ESP are explored in the “a perceived responsibility of a change agent” category. The category revealed that the participants could recognize the responsibilities of a leader in the ups and downs of an educational change project. It is suggested to use ESP as a faculty development program to train educational leadership for change.

## Supplementary Information

**Additional file 1:** **Appendix 1.** The interview questions.

## Data Availability

The datasets used and/or analyzed during the current study are available from the corresponding author upon reasonable request.
