# Supplementary material for: A developmental pathway toward leadership for educational change: the Educators’ experiences of the educational scholar program
Source: BMC Med Educ. 2023 Jan 16;23:30. doi: 10.1186/s12909-023-04015-8 (PMC9843881; doi:10.1186/s12909-023-04015-8)
Supplement: Supplementary file 1 — Additional file 1: Appendix 1. The interview questions. [file 12909_2023_4015_MOESM1_ESM.docx]

Appendix 1:

| **Appendix 1**- The interview questions | |
| --- | --- |
| Antecedents | - The ESP is optional, what made you apply? - What factors informed this decision? |
| Process | - Would you please describe your scholarship project in the training? |
|  | - Please describe your experience about contributing ESP and implementing SoTL project? |
|  | - In what ways did ESP impact your experience? - What positive and challenging subjects have you experienced in ESP? |
| Outcomes | - In what ways did ESP change how you think about your career goals? - In what ways did ESP impact your experience? |
